# Cognitive functioning in meningioma patients: a systematic review

**DOI:** 10.1007/s11060-016-2115-z

**Published:** 2016-04-05

**Authors:** Ikram Meskal, Karin Gehring, Geert-Jan M. Rutten, Margriet M. Sitskoorn

**Affiliations:** Department of Cognitive Neuropsychology, Tilburg University, Warandelaan 2, PO Box 90153, 5000 LE Tilburg, The Netherlands; Department of Neurosurgery, St Elisabeth Hospital, Tilburg, The Netherlands

**Keywords:** Meningioma, Cognition, Neuropsychological, Attention, Memory, Executive functions, Neurosurgery, Radiotherapy

## Abstract

This systematic review evaluates relevant findings and methodologic aspects of studies on cognitive functioning in meningioma patients prior to and/or following surgery with or without adjuvant radiotherapy. PubMed and Web of Science electronic databases were searched until December 2015. From 1012 initially identified articles, 11 met the inclusion criteria for this review. Multiple methodological limitations were identified which include the lack of pre-treatment assessments, variations in the number and types of neuropsychological tests used, the normative data used to identify patients with cognitive deficits, and the variety of definitions for cognitive impairment. Study results suggest that most of meningioma patients are faced with cognitive deficits in several cognitive domains prior to surgery. Following surgery, most of these patients seem to improve in cognitive functioning. However, they still have impairments in a wide range of cognitive functions compared to healthy controls. Suggestions are given for future research. Adequate diagnosis and treatment of cognitive deficits may ultimately lead to improved outcome and quality of life in meningioma patients.

## Introduction

As a result of increasingly effective disease management, patients with brain tumors have better survival rates. This prompts a different approach towards health care. Instead of considering survival as the sole endpoint, quality of survival is also considered [1]. The assessment of health related quality of life (HRQoL) and cognitive function has become increasingly recognized as an important outcome measure in brain tumor research. Cognitive functioning has a significant impact on HRQoL, and could even be a predictor of HRQoL [2].

To date, most studies on cognitive functioning in brain tumor patients have focused on glioma patients. Less is known about cognitive functioning in meningioma patients and the impact of surgery and/or (adjuvant) radiotherapy [3–10]. Rapidly growing tumor types such as high-grade gliomas typically lead to more cognitive impairment than slowly growing tumors such as meningiomas [11, 12]. However, even meningiomas can cause cognitive deficits by putting pressure on brain tissue [13]. These tumors often grow to a considerable size before clinical symptoms appear because of the plastic potential of the brain [14–17].

The objective of this systematic review was to evaluate the available data and the quality of studies on cognitive impairment in meningioma patients prior to and/or following treatment, and to document potential changes in cognitive dysfunction due to treatment (i.e., surgery with or without adjuvant radiotherapy). We also reviewed methods used to evaluate cognitive function in meningioma patients, and make recommendations for future studies.

## Methodology (systematic review)

### Inclusion criteria

This systematic review included peer-reviewed research articles on cognitive functioning in adult patients with meningioma prior to and/or following surgery with or without adjuvant radiotherapy, as assessed with neuropsychological tests.

### Search strategy

Searches were conducted using the electronic databases of PubMed (MEDLINE) and Web of Science (Web of Knowledge). For each database, searches included the terms: mening* or *brain* or *cerebral* or *cranial* (title/abstract, topic), in addition, an ‘and’ condition was specified for the following 2 groups of terms: (1) neuropsycholog* or cognit* or neurocognit* or attention* or *memory* or executive function* (title), (2) tumor* or tumour* or neoplasm* (title).

Searches were limited to adult human-beings and peer-reviewed original research papers written in English. In addition, results of studies that examined cognitive functioning in groups of brain tumor patients were also included if separate analyses were done for meningioma patient groups. Studies without objective measures of cognitive function as assessed with neuropsychological tests were excluded. Studies that used very short screening tests, such as Mini-Mental State Examination (MMSE) and 3MS examination (modified MMSE) were included, but are only briefly discussed. There were no restrictions on publication dates, and the final searches were done in December 2015.

### Study selection process

In total, 2205 article citations (i.e., 873 in PubMed + 1332 in Web of Science) were found and downloaded into EndNote [18]. These were scanned using EndNote for duplicates, and 1193 were deleted, yielding a final total of 1012 articles.

Then, the titles of these articles were sifted to exclude all articles that did not meet the objectives of this review, which resulted in the removal of 886 articles. This first sift resulted in 126 articles for which abstracts and/or full text articles were assessed in detail. Subsequently, 115 (out of 126) articles were rejected because they did not meet the inclusion criteria, were conference presentations or case reports. The remaining 11 articles were examined jointly by 2 reviewers and remain included for this review.

## Results

Table 1 summarizes the 11 studies that evaluated cognitive functioning in meningioma patients prior to and/or following treatment. In this section, results from studies including pre-operative and post-operative cognitive assessments are discussed. The effects of adjuvant radiotherapy on cognitive outcomes are discussed in a subsequent section. Potential associations of cognitive impairment with tumor location and other factors are presented in Box 1.

**Table 1:** Studies on cognitive functioning in meningioma patients

Study	Patient group	Control group	Treatment	NP tests/domains	Timing of assessment(s)	Definition of CI	Statistics	Relevant results prior to treatment	Relevant results following treatment
Tucha [4]	54 ST frontal MGM Grade^1^ = NR M age = 57.8 (SD = 1.5) M vol = 79.3 cm (SD = 11.3)	54 matched HC M age = 57.9 (SD = 1.5)	Surgical removal	VerM, VisM, Att, EF, VC	2–3 days before surgery, 4–9 months after surgery	None	Non parametric tests, Ipsative scores^2^, Spearman rank corr	Sign lower mean raw scores, longer reaction times, or higher error rates on measures of WM, Att, and EF	Sign lower mean raw scores, longer reaction times, or higher error rates on Att and EF. Sign improvements on measures of (immediate recall) VisM and Att
Tucha [10]	33 ST MGM Grade = NR M age = 72.8 (SEM = 0.9) M vol = NR 7 tumors diameter ≤ 4 cm 26 tumors diameter > 4 cm	20 HC M age = 73.1 (SEM = 1.0)	Surgical removal	Same as in [4]	Before surgery, 3–6 months after surgery	None	(Non) parametric tests	Sign lower mean raw scores, longer reaction times, or higher error rates on measures of WM, (short-term) VisM, Att, and EF	Sign improvements on measures of (short-term) VisM and Att. Comparable post-op cogn status to cogn functioning of elderly HC, except for WM
Meskal [20]	68 ST and IT MGM Grade I M age = 55.7 (range = 36–74) M vol = NR Maximum diameter = 4.38 cm	Norms based on healthy American population [22] (N = 1,069)	Surgical removal	Mem, PsyMo, RT, Att, CogFlex, ProcSp, EF (computer tests)	1 day before surgery, 3 months after surgery	Standard scores ≥1.5–2 SD below norm	T-tests, Mc Nemar’s tests, Pearson product-moment corr	Sign lower standard scores on all cogn domains. 47/68 pts (69 %) low or very low on 1 or more cogn domains	Sign improved mean standard scores for all cogn domains, except PsyMo and RT. Sign lower mean standard scores for all cogn domains. 27/62 pts (47 %) low or very low on 1 or more cogn domains
Yoshii [7]	34 MGM Grade = NR Right-sided: M age = 64 (SD = NR) Left-sided: M age = 59 (SD = NR) M vol = NR 83 glioma	Normative healthy population values from manual (reference: could not be retrieved)	Surgical removal	3MS test	Before surgery and/or within 1 month after surgery	Subnormal cogn function = 3 MS score <85	NR	Subnormal cogn function. No differences in cogn function between left-sided and right-sided MGM	Normalization only in right-sided group, not in left-sided group
Koizumi [19]	10 ST MGM Grade I: (n = 9), grade II: (n = 1) M age = 68.1 (SD = 13.1) M vol = 89.1 ml (SD = 51.8)	Normative healthy population values (reference: NR)	Surgical removal	MMSE IZM-SPECT	Within 4 weeks before surgery, 3 months after surgery	MMSE score ≤23	NR for MMSE data, T-tests for IZM-SPECT data	Mean MMSE scores = 19.9 (SD = 11.4), ranging from 2 to 30	Sign improvement of cogn function, mean MMSE score = 26.5 (SD = 3.8)
Van Niewenhuizen [6]	21 untreated MGM, wait-and-scan approach Grade I M age = 63.4 (SD = 13.5) M vol = 6.3ml (SD = 6.5)	21 normative matched (age, gender, education) HC from MAAS [26] (N = 2,000), M age = 62.4 (SD = 12.9)	None	VerM, WM, Att, EF, PsyMo, InfPro	Once	Z-scores ≥1.5 SD below norm	Mann-Whitney U-tests, Kendall’s Tau	Sign lower mean Z-scores on WM and PsyMo, sign better mean Z-scores on VerM	NAp
Steinvorth [27]	40 ST MGM Grade = NR M age = 55 (SD = 14) M vol = NR 1 total resection: (n = 16), 1 partial resection: (n = 8), ≥ 2 resections: (n = 6), no resection: (n = 10)	Normative healthy population values from manuals	FSRT	VerM, VisM, Att, IQ	1 day before FSRT, within 24 hrs after first fraction, at end of FSRT, 6 weeks, 6 and 12 months after FSRT	None	(Non) parametric tests	Sign lower mean pct scores on Att/InfPro	After first fraction: transient decline in Mem and improvements in Att. During further follow-up: no deteriorations, but further improvements in Mem and Att
Van Niewenhuizen [8]	36 ST MGM Grade I 18 RTx+: surgery and RTx M age = 63.3 (SD = 10.6) M vol = 39.4 ml (SD = 43.5) 18 RTx-: surgery only M age = 62.6 (SD = 11.8) M vol = 23.5 ml (SD = 19.3)	18 normative matched (age, gender, education) HC from MAAS [26] (N = 2,000) M age = NR	Surgical removal with (RTx+) /without (RTx-) adjuvant RTx	Mem, Att, EF, Perc	≥1 year after surgery	None	Chi-square tests, T-tests	NAp	No sign differences in mean standard scores on all cogn domains between RTx- and RTx+. Sign lower mean standard scores on all cogn domains in RTx-. No comparisons were made for cogn functioning between RTx+ group and HC
Krupp [9]	91 ST MGM Grade I M age = 56 (SD = 10) M vol = NR	Normative healthy population values from manuals	Surgical removal	Tests of Att, IQ	10–18.5 months after surgery	None	T-tests, Chi-square tests, ANOVA, Spearman rank corr, Regression analyses	NAp	Negative corr between age and Att in pts > 55 yrs, as well as with IQ factors verbal knowledge, technical ability, and word fluency
Dijkstra [5]	89 ST MGM Grade I M age = 58.6 (SD = 12.1) M vol = 46.1 ml (SD = 51.8)	89 normative matched (age, gender, education) HC from MAAS [26] (N = 2,000) M age = 58.3 (SD = 13.3)	Surgical removal with (n = 22)/ without adjuvant RTx (n = 67)	VerM, WM, Att, EF, PsyMo, InfPro	≥1 year after surgery	Z-scores ≥1.5 SD below HC mean	T-tests, Multiple regression analyses	NAp	Sign lower mean Z-scores on all domains, except for Att
Waagemans [2]	89 ST MGM Grade I M age = 58.4 (SD = 13.2) M vol = 46.1 ml (SD = 51.8)	89 normative matched (age, gender, education) HC from MAAS [26] (N = 2,000) M age = 58.3 (SD = 13.3)	Surgical removal with (n = 22)/without adjuvant RTx (n = 67)	Same as in [5]	≥1 year after surgery	Z-scores ≥1.5 SD below HC mean	T-tests, Multiple regression analyses	NAp	Same population as in [5]. Similar findings on cogn functioning as in [5]

*ANOVA* analyses of variance, *Att* attention, *CI* cognitive impairment, *CogFlex* cognitive flexibility, *Cogn* cognitive, *Corr* correlation, *EF* executive function, *FSRT* fractioned stereotactic radiotherapy, *GL* glioma, *HC* healthy controls, *IZM-SPECT* ¹²³I-iomazenil (*IMZ*) single-photon emission computed tomography (SPECT) imaging, *IT* infratentorial, *M* mean, *MAAS* Maastricht Aging Study [26], *Mem* memory, *MGM* meningioma, *MMSE* mini-mental state examination, *NAp* not applicable, *NR* not reported, *Pct* percentile, *Perc* perception, *Post-op* post-operative, *ProcSp* processing speed, *PsyMo* psychomotor speed, *Pt*(s) patient(s), *RT* reaction time, *RTx* radiotherapy, *SD* standard deviation, *Sign* significant, *ST* supratentorial, *VC* visuoconstructive abilities, *VerM* verbal memory, *VisM* visual memory, *Vol* tumor volume, *WM* working memory, *3MStest* modified mini-mental state exam

^1^Grade based on the World Health Organization (WHO) classification

^2^These results were not reported in the review. Ipsative scores = substraction post-operative testscores from pre-operative test scores

**Box 1:** Tumor location and other relevant factors related to cognitive performance prior to and/or following treatment

Relevant factors	Relevant findings	Study
Tumor location	No sign differences in cognitive status between lateralization groups prior to and following surgery	Tucha [4]
	Sign differences in changes over time between lateralization groups, mainly on attentional functions. Left-sided (n = 22) MGM improved sign on flexibility and shifting. Right-sided (n = 21) MGM improved sign on variety of attentional functions	
	Sign effect of frontal MGM on pre-operative and post-operative cognitive status. Prior to surgery; falx cerebri (n = 14) performed sign better on figural fluency than frontobasal (n = 19) and convexity (n = 17) MGM. Following surgery; frontobasal (n = 19) and falx cerebri (n = 14) MGM performed sign better on divided attention and figural memory than convexity (n = 17) MGM	
	Sign differences between localization groups for various cognitive domains. Convexity (n = 17) MGM: only improvement on flexibility and shifting (attentional/executive functions), frontobasal (n = 19) MGM: improvement on a broader range of attentional/executive functions after surgery. Pts with falx cerebri (n = 14) MGM improved on various cognitive domains	
	No sign differences in cognitive status between lateralization groups prior to and following surgery	Meskal [20]
	No sign associations between tumor lateralization and cognitive improvement over time	
	No sign differences in pre-operative or post-operative cognitive functioning based on tumor localization, except for complex attention: sign better performance for infratentorial (n = 7) as opposed to supratentorial (n = 61) tumors	
	No sign associations between tumor localization (skull base, convexity, and convexity/falx) and cognitive improvement over time	
	Cognitive function normalized in right-sided (n = 17) MGM following surgery. Left-sided (n = 17) MGM did not normalize or improve	Yoshii [7]
	No statistical tests were conducted in this study: no clear conclusions can be drawn	
	No reports on specific localization or lateralization effects on cognitive functioning	Koizumi [19]
	Based on data in a table; 3 pts with very low scores (<10) on MMSE before surgery, suffered from convexity (n = 4) MGM. These pts improved substantially after surgery, but still had the lowest scores on MMSE (≤ 23), compared with other localization groups	
	No clear associations of memory functions with localization before FSRT (no data reported)	Steinvorth [27]
	No clear lateralization effects before and after FSRT	
	Pts with left-sided (n = 37) MGM performed sign worse on verbal memory compared to right-sided (n = 25) MGM	Dijkstra [5]
	Lower cognitive performance in skull-base (n = 24) MGM on verbal memory, information processing, and psychomotor speed compared to convexity (n = 28) MGM. Not clear as to whether theses analyses were done in smaller subgroups of the study sample	
Epilepsy	Sign negative correlation between epilepsy burden and executive functioning, primarily due to AEDs use, not to epileptic seizures	Dijkstra [5]
	Sign impaired cognitive functioning also in pts who did not use AEDs (n = 66) compared with HC	
	Comparable HRQoL in pts to that in HC	Waagemans [2]
	HRQoL worse in pts with cognitive deficits and pts who use AEDs, irrespective of seizure control	
Mood	No sign correlation between anxiety and cognitive domains, negative correlation between depression and 6/7 cognitive domains prior to surgery (n = 60 out of 68)	Meskal [20]
	Negative correlation between anxiety and attention, negative correlation between depression, memory and attention following surgery (n = 52 out of 62)	
	Sign improvement toward a positive mood from baseline (no data reported) up to 6 weeks after follow-up of FSRT	Steinvorth [27]
	No correlations were investigated	
Quality of life	RTx+ pts lower HRQoL than RTx- pts	Van Nieuwenhuizen [8]
	No sign differences in HRQoL between RTx- pts and HC. After correction for duration of disease, no sign differences in HRQoL between both MGM groups	
	No comparisons were made for HRQoL between RTx+ pts and HC	
	No sign differences between pts and HC on 7/8 HRQoL scales	Waagemans [2]
	Impaired executive functioning had a direct negative relationship with other cognitive domains (information processing, verbal memory, psychomotor speed, and attention), and an indirect negative relationship with HRQoL	
Other factors	IZM-SPECT images showed recovered binding potential of IZM following surgery	Koizumi [19]

*AEDs* anti-epileptic drugs, *FSRT* fractioned stereotactic radiotherapy, *HC* healthy controls, *HRQoL* health-related quality of life, *IZM-SPECT* ¹³³I-iomazenil (IMZ) single-photon emission computed tomography (SPECT) imaging. *MGM* meningioma, *MMSE* mini-mental state examination, *Pts* patients, *RTx* radiotherapy. *Sign* significant

### Cognitive functioning in meningioma patients prior to and/or following surgery

Cognitive functioning prior to treatment was examined in 5 studies with a total of 199 meningioma patients eligible for surgery [4, 7, 10, 19, 20] (see Table 1). Overall, in these studies, cognitive functioning has been found impaired. Most commonly affected domains were memory, attention, and executive functions. Cognitive functioning following surgery was investigated in 7 studies including a total of 302 meningioma patients [4, 7–10, 19, 20] (see Table 1). All studies, except 2 [8, 9], started with a pre-operative assessment. Pre-operative assessments allow to determine possible effects of surgery on cognitive performance. Only 2 [4, 20] of the 5 studies with a repeated (pre-and post-operative) assessment of cognitive function controlled for the influence of practice effects. In general, all studies showed significant improvements following surgery in cognitive functioning, mostly on memory, attention, and executive function. There was no consistency in results across studies with regard to the cognitive domains that did not improve after surgery. However, despite cognitive improvements, all studies (including those without pre-operative assessment) demonstrated that patients (still) had significantly lower scores in various cognitive domains after surgery, compared to healthy controls. For studies including a pre-and post-operative assessment (mean interval between 2 assessments ranging from 3 to 9 months), no clear conclusions can be drawn on the effect of time since surgery on the post-operative cognitive outcome. Severity data (e.g., effect sizes, incidences) were not available for most of them, due to differing populations.

In particular, Tucha and colleagues [4] found significant pre-operative impairments in patients with frontal meningiomas (N = 54) on measures of working memory, attention, and executive functions (lower mean raw scores, longer reaction times, or higher error rates), compared to healthy controls (matched for age, gender, educational level, handedness, and intelligence). After surgery, significant improvements were observed on measures of memory and attention. However, despite these significant improvements, patients’ post-operative status remained significantly impaired in attention and executive functions, compared to healthy controls who were retested over the same intervals. According to the authors, only the better post-operative performance in figural memory (immediate recall) could be partly explained by practice effects by comparing the test results with the healthy control group. Note that the authors classified flexibility and shifting as subdomains of attention. However, these measures can also be considered as components of executive functioning [21].

In addition, Tucha and colleagues [10] conducted a study with *elderly* meningioma patients (N = 33). These patients showed significant pre-operative impairments on measures of working memory, short-term figural memory, attention, and executive functions (lower mean raw scores, longer reaction times, or higher error rates), compared to healthy controls in the same age-range. After surgery, significant improvements were observed on measures of memory and attention, with the exception of working memory. In this study, patients’ post-operative cognitive status corresponded with the cognitive functioning of the healthy control group (except for working memory). Because the healthy controls were only tested once, it was not possible to rule out practice effects, which may have masked lower performance in the elderly meningioma patients. See the above-mentioned note regarding the classification of cognitive domains by these authors. It was not reported if there was overlap in patients between these 2 studies by Tucha and colleagues; a certain amount of overlap between the patient samples seems possible [4, 10].

In a recent study by Meskal and colleagues [20], meningioma patients (N = 68) had significantly lower mean pre-operative and post-operative standard scores on measures of memory, psychomotor speed, reaction time, complex attention, cognitive flexibility, processing speed, and executive functioning, compared to (American) normative data as provided by the Central Nervous System Vital Signs battery (i.e. CNS VS), a brief (30 min) computerized battery of neuropsychological tests [22]. Forty-seven out of 68 patients (69 %) scored low or very low on 1 or more cognitive domains. After surgery, significant improvements were observed on all cognitive domains, with the exception of psychomotor speed and reaction time. Twenty-seven out of 62 patients (47 %), scored low or very low on 1 or more cognitive domains after surgery.

The 3MS test used in a study by Yoshii and colleagues [7] showed a subnormal function (mean 3MS score < 85) in 34 meningioma patients pre-operatively. Cognitive function normalized after surgery only in patients with right-sided (n = 17) meningioma (post-surgery mean 3MS score > 85). Note that the authors have chosen for a more stringent cut-off of 85 instead of 77/78, which is generally used as cut-off for cognitive impairment [23]. In addition, patients were tested within 1 month after surgery, which is a very short follow-up time that may identify (more severe) transitory cognitive problems instead of persistent cognitive deficits in left-sided meningioma patients. Furthermore, it was not clearly described by the authors why some patients had only 1 assessment (i.e., prior to, or following surgery), and other patients were assessed twice with the 3MS test (prior to, and following surgery).

Another study, by Koizumi and colleagues [19], evaluated cognitive dysfunction with the MMSE in meningioma patients (N = 10) who also underwent ^123^I-Iomazenil (IMZ) single-photon emission computed tomography (SPECT) imaging. The mean pre-operative MMSE scores were 19.9 ± 11.4; ranging from 2 to 30. The MMSE cut-off points for normal, mild, moderate, and severe cognitive impairment were not described by the authors. Based on the MSSE cut-off levels application by Folstein and colleagues [24], 3 patients had moderate to mild cognitive impairment (scores on MMSE ranging from 20 to 25), and 3 patients had severe cognitive impairment (scores ranging from 2 to 5); 4 of them had scores of 29–30. Overall, 6 patients scored above the cut-off point of 23. After surgery, a significant improvement in cognitive function (mean post-surgery MMSE: 26.5 ± 3.8) was found. Seven of the 10 patients scored above the cut-off of 23 on the MMSE, which suggests ‘normal’ cognitive functioning in those patients. Note that screening tests such as the MMSE and 3MS are not sensitive enough to discriminate between mild cognitive impairment and normal cognitive functioning [25].

Van Nieuwenhuizen and colleagues [6] found significantly lower mean Z-scores in patients with a wait-and-scan policy (N = 21) on measures of psychomotor speed and working memory, compared to normative matched healthy controls from the Maastricht Aging Study (i.e., MAAS [26]). Note that this study was conducted in a specific group of meningioma patients, in which the tumor was small, growing slowly, and was not causing symptoms or if surgery carried too many risks, particular for older patients who are more vulnerable to develop complications after surgery due to their medical condition.

Steinvorth and colleagues [27] included 10 patients admitted only for fractioned stereotactic radiotherapy (FSRT) instead of surgery. However, the authors did not report cognitive results. Note that the patients who were included in the studies by Van Nieuwenhuizen and colleagues [6] and Steinvorth and colleagues [27] were substantially different (e.g., smaller tumor volumes, inoperable meningiomas after subtotal resection or recurrence) from those patients who were admitted for surgical treatment. Therefore, the results of the aforementioned 2 studies cannot be generalized to the general population of meningioma patients admitted for surgery.

In another study by Van Nieuwenhuizen and colleagues [8] in which some (n = 18) meningioma patients were tested only after surgery and not before, significantly lower mean standard scores were found on a number of verbal memory subtests, compared to normative healthy controls. The authors concluded that these patients had significantly lower cognitive functioning than healthy controls. Attention and executive function were not impaired in these patients. The patients of this study were compared with patients (n = 18) who received adjuvant radiotherapy after surgery (RTx+). The results of the latter patient group are discussed in the section on effects of adjuvant radiotherapy. It should be noted that although overlap in patients between this study and the above-mentioned study of Van Nieuwenhuizen cannot be ruled out, this is not likely since the study in patients who had already undergone surgery [8] preceded the study in patients in whom surgery was not performed [6].

Similar to the aforementioned study, Krupp and colleagues [9] investigated cognitive functioning after surgery without a pre-operative assessment in 91 patients. Compared with published normative population values, major deficits in attention appeared in patients of approximately 55 years of age, worsening in patients with increasing age. Significant negative correlations were found between age and attention performance in patients older than 55, as well as with the intelligence factors verbal knowledge, technical ability, and word fluency. No such correlation was found for reasoning and age. Since no pre-treatment assessment was available in the aforementioned 2 studies, the specific effects of the brain tumor or surgery on cognitive performance cannot be determined.

### Cognitive functioning in meningioma patients: effects of adjuvant radiotherapy

Three studies investigated cognitive functioning in meningioma patients who had undergone radiotherapy after surgery [2, 5, 8]. These studies described the same [2, 5] or an overlapping ([8]) patient sample, but investigated different types of research questions. In these studies, patients in whom the tumor could only be partially resected and patients with a recurrence after surgery received adjuvant radiotherapy.

The study by Van Nieuwenhuizen and colleagues [8] investigated the exclusive effects of adjuvant radiotherapy after surgery by comparing patients who had surgery only (RTx−) with patients who had surgery and adjuvant radiotherapy (RTx+). The authors found no significant differences in mean standard scores on all cognitive measures (memory, attention, executive function, and perception) between RTx− (n = 18) and RTx+ (n = 18) patients (which may be patients with different tumor characteristics). No comparisons were made for cognitive functioning between the RTx+ group and healthy controls. In this study, additional radiotherapy did not have deleterious effects on cognitive functioning. The studies by Dijkstra and colleagues [5] and Waagemans and colleagues [2] did not differentiate between the effects of surgery and/or radiotherapy. In the study by Dijkstra and colleagues [5], patients (N = 89) showed significantly lower mean Z-scores on measures of verbal memory, visual memory, working memory, information processing, psychomotor speed, and executive function (most impaired), compared to normative matched healthy controls (from MAAS [26]). No significant differences were found for attention. Note that the proportions of patients with cognitive deficits (defined as 1.5 SD below the mean of a matched control group) was not reported by these authors. The study by Waagemans and colleagues [2] focused on HRQoL and reported similar findings on cognitive functioning in meningioma patients (N = 89) as in the study by Dijkstra and colleagues [5]. A common limitation of the aforementioned studies was an absence of a pre-treatment assessment of cognitive functioning. Also noteworthy is the large standard deviation (SD) of tumor volumes in these studies.

Only 1 study [27] investigated the effects of FSRT following surgery in meningioma patients (n = 30). In this study, cognitive function was evaluated before and after FSRT. Patients had normal mean percentile scores, except for a slow information processing speed prior to radiotherapy. After the first fraction, a transient decline in memory and, at the same time, improvements in attentional functions were observed. No deteriorations were seen during the further follow-up, but further increases in memory and attention were observed. Note that the improvement in attention was considered as a practice effect, since a comparable improvement was also observed in a control group, included in an earlier report by these authors [27].

## Conclusion and recommendations

This systematic review provides an overview of studies investigating cognitive functioning in meningioma patients prior to and/or following surgery with or without adjuvant radiotherapy.

Drawing conclusions from studies and comparison of results between them were complicated by several methodological limitations, such as a lack of pre-treatment assessments, variations in the number and types of neuropsychological tests used, definitions of cognitive impairment, quality of normative data, and absence of control for practice effects.

Specific effects of treatment cannot be determined in the absence of an assessment before treatment. The number of patients with above average cognitive abilities before treatment may be underestimated. Patients may have a functional decline, but still perform within normal ranges on cognitive tests. In addition, cognitive deficits that have been present before treatment may be unjustly attributed to surgery. None of the studies described the presenting symptoms of the meningioma patients included. Therefore, it is not clear if cognitive complaints were present at neuropsychological assessment. As the cognitive status of patients with incidentally-detected meningiomas is likely to differ from that in patients presenting with cognitive complaints, it is not clear as to whether the samples were representative of all meningioma patients.

In addition, the number and types of neuropsychological tests used, varied across studies and complicated comparison of results. For example, 8 studies [2, 4–6, 8–10, 27] tested patients with a traditional neuropsychological battery that consisted of 2 to 12 paper-and-pencil tests. One study used a computerized screening battery (i.e., CNS VS [22]) consisting of 7 neuropsychological tests [20]. Two studies [7, 19] used very global screening tests (i.e., MMSE and 3MS), that are known to have a low sensitivity and are not useful for screening for subtle cognitive impairment [25].

Quality of normative data also differed between studies, 2 studies included their own healthy control group matched on different variables [4, 10], 4 studies used normative matched data from 18 to 89 healthy controls from the Maastricht Aging Study (MAAS [26]) [2, 5, 6, 8], and 5 studies used (published) normative healthy population values as provided by the test (manual) [7, 9, 19, 20, 27].

Further, definitions used to classify patients as having cognitive impairment differed across studies. Three studies [2, 5, 6] used Z-scores and defined individual cognitive impairment as 1.5 SD below the mean of a matched control group. One study [20] defined standard scores of 1.5 and 2 SD below the mean of a normative control group as cognitive impairment. Five studies [4, 8–10, 27] did not use a definition of individual cognitive impairment. None of the studies reported a cut-off for (general) cognitive impairment on the number of tests required to be in an impaired range. Only 1 study [20] reported on the incidence and severity of cognitive impairment.

Finally, only 2 [4, 20] of the 5 studies with a pre-and post-treatment assessment considered the influence of practice effects on improved cognitive function after repeated testing by including a (matched) control group that was tested twice with the same test battery. The computerized test battery CNS VS is assumed to be suitable for repeated testing because of the random presentation of stimuli [20, 22]. However, despite the chance that a patient gets the same stimuli twice is negligible, there still could be a learning effect of the battery in general, also known as test-wiseness [28]. The patient knows what to expect the second time. Thus, longitudinal studies without consideration of practice effects may report better results due to repeated exposure to neuropsychological testing. Practice effects may therefore mask cognitive decline or stability.

Moreover, many studies reviewed here lacked a clear description of statistical testing, or only very basic statistical analyses were conducted. For example, some studies only performed univariate analyses where no correction for potential other differences between groups was applied when comparing effects of tumor localization (among groups).

To overcome some of the methodological issues described, we recommend using a test battery with a wide range of neuropsychological tests that is sensitive enough for identifying subtle cognitive impairment in patients and suitable for serial repetition. In addition, a pre-treatment assessment, a sufficiently large sample size to conduct (multivariate) analyses, a uniform definition of cognitive impairment, and appropriate quality of normative data are suggested.

Despite these limitations, the studies in this review demonstrate that meningioma patients have impaired cognitive functioning prior to treatment. In general, most commonly affected domains were memory, attention, and executive functions. Surgery generally had a beneficial effect on cognitive function. A significant improvement in cognitive functioning was found 3 to 9 months following surgery, mostly on memory, attention, and executive function. Cognitive performance still remained below normal however. There is no consistency across studies about the domains that did not improve after surgery. In the one study on adjuvant radiotherapy, no additional deleterious effects on cognitive functioning at least 1 year after surgery were found. Two other studies found that the use of AEDs negatively affects cognitive functioning and HRQoL.

Mixed findings were reported with respect to effects of lateralization and localization of the tumor on cognitive impairment. In most studies, associations between cognitive functioning and other tumor characteristics (i.e., volume, edema) were not observed [2, 4, 5] or could not be made because of the small sample sizes in the studies [6]. Other factors that are known to have a relation to cognitive performance prior to and/or following treatment, such as epilepsy, mood, and HRQoL were not systematically investigated across studies.

There is evidence to conclude that meningioma patients are faced with cognitive dysfunction in several cognitive domains before and (slightly less) after treatment. Clinicians should be aware of these deficits. Researchers should employ more rigorous methodologies. Better awareness, early diagnosis and treatment of cognitive deficits may improve outcome and quality of life in this patient population.
